# Bidirectional Links Between Sleep and Marital Quality: An Eight-Year Longitudinal Study of Older Couples

**DOI:** 10.1093/geroni/igaf122.3549

**Published:** 2025-12-31

**Authors:** Hanamori Skoblow, Eunjin Tracy, Megan Gilligan

**Affiliations:** University of Missouri, Columbia, Missouri, United States; University of Missouri, Columbia, Missouri, United States; University of Missouri, Columbia, Missouri, United States

## Abstract

The Model of Dynamic Association Between Relationship Functioning and Sleep positions sleep as a fundamentally dyadic process, underscoring the need to study sleep within its relational context and highlighting the value of longitudinal, dyadic research to better understand interconnected pathways between sleep and close relationships. Although sleep problems and the salience of marital ties increase with age, few studies have tested links between partners’ sleep and marital quality with older samples. Therefore, we investigated longitudinal and bidirectional within- and between-person associations between sleep and marital quality in couples aged 50 + (M = 63.12; SD = 7.87). Using dyadic random intercept cross-lagged panel models, we tested associations between multiple domains of sleep (i.e., insomnia and subjective sleep quality) and marital quality (i.e., support and strain) over three waves of data spanning eight years in a nationally representative sample of 2,343 couples from the English Longitudinal Study of Ageing. Women’s reports of greater spousal strain predicted increases in their own insomnia symptoms and decreases in their subjective sleep quality over time. Additionally, women’s higher-than-average perceptions of spousal support predicted men’s later improvements in subjective sleep quality. No significant cross-lagged effects emerged between insomnia and spousal support, nor did we detect partner effects between spousal strain and subjective sleep quality. These findings suggest that women’s reports of marital quality are more consequential for both partner’s sleep, emphasizing the importance of considering gendered processes and interpersonal dynamics when examining the reciprocal links between sleep and relationship functioning across time.

